# An Appraisal of the Maternal Mortality Decline in Nepal

**DOI:** 10.1371/journal.pone.0019898

**Published:** 2011-05-26

**Authors:** Julia Hussein, Jacqueline Bell, Maureen Dar Iang, Natasha Mesko, Jenny Amery, Wendy Graham

**Affiliations:** 1 Immpact, University of Aberdeen, Aberdeen, Scotland; 2 United Mission to Nepal, Kathmandu, Nepal; 3 Department for International Development Nepal, Kathmandu, Nepal; 4 Department for International Development London, London, United Kingdom; Institute of Clinical Effectiveness and Health Policy, Argentina

## Abstract

**Background:**

A decline in the national maternal mortality ratio in Nepal has been observed from surveys conducted between 1996 and 2008. This paper aims to assess the plausibility of the decline and to identify drivers of change.

**Methods:**

National and sub-national trends in mortality data were investigated using existing demographic and health surveys and maternal mortality and morbidity surveys. Potential drivers of the variation in maternal mortality between districts were identified by regressing district-level indicators from the Nepal demographic health surveys against maternal mortality estimates.

**Results:**

A statistically significant decline of the maternal mortality ratio from 539 maternal deaths to 281 per 100,000 (95% CI 91,507) live births between 1993 and 2003 was demonstrated. The sub-national changes are of similar magnitude and direction to those observed nationally, and in the terai region (plains) the differences are statistically significant with a reduction of 361 per 100,000 live births (95% CI 36,686) during the same time period.

The reduction in fertility, changes in education and wealth, improvements in components of the human development index, gender empowerment and anaemia each explained more than 10% of the district variation in maternal mortality. A number of limitations in each of the data sources used were identified. Of these, the most important relate to the underestimation of numbers of deaths.

**Conclusion:**

It is likely that there has been a decline in Nepal's maternal mortality since 1993. This is good news for the country's sustained commitments in this area. Conclusions on the magnitude, pattern of the change and drivers of the decline are constrained by lack of data. We recommend close tracking of maternal mortality and its determinants in Nepal, attention to the communication of future estimates, and various options for bridging data gaps.

## Introduction

Nepal has a population of 29 million people and is divided into three ecological zones: mountain, hill and terai (plains). There are 75 administrative districts and 16% of the population live in urban areas. It is a relatively poor country with a human development index of 0.6 (ranking 144^th^ out of 182 countries) and large gender disparities [1]. Civil unrest over the last two decades has occurred. The political instability and diverse geographical terrain have been key challenges for socio-economic development.

Nevertheless, Nepal Demographic and Health Surveys (NDHS) published in 1996, 2001 and 2006 have shown declining fertility rates from 4.6 to 3.1. Modern contraceptive use among currently married women in Nepal has increased from 26% to 44.2%. There are indications that access to maternity services is also improving. Births with health professionals have doubled from 9.0% to 18.7% and Caesarean sections in the population have nearly tripled to 2.7%. Antenatal care coverage is now 73.7%. Reductions in maternal mortality from 539 to 281 per 100,000 live births were noted between the 1996 and 2006 reports. Neonatal mortality has fallen from 49.9 to 33.0 per 1,000 live births [2]–[4]. Despite these improvements, there remain disparities between urban and rural settings and richer and poorer sectors of society [5].

Safe motherhood has been a national priority programme within the health sector for the last 15 years. Major government health policies and plans have been revised in line with millennium development goals, global strategies on skilled birth attendants and new born care, equity aims and poverty alleviation. Implementation of Nepal's national safe motherhood programme was supported by the United Kingdom's Department for International Development (DFID) through the Government of Nepal. The programme received technical assistance from Options via the Nepal Safer Motherhood Project (NSMP) from 1997 to 2004, and through the Support to the Safe Motherhood Programme (SSMP) between 2005 and 2010. In 2008/9, the Maternal Mortality and Morbidity Study (MMMS) was conducted by the Nepal government's Family Health Division, with technical support from Options [6].

Recent global estimates of maternal mortality suggest declines. The World Health Organization reports a fall of 34% since 1990, to a current global level of 260 maternal deaths per 100,000 live births, broadly agreeing with independent estimates from the Institute of Health Metrics and Evaluation [7], [8]. For Nepal, estimates of maternal deaths per 100,000 live births from the Institute of Health Metrics and Evaluation [8] were 471 (CI 290–722) in 1990 and 240 (CI 149–370) in 2008, while the World Health Organization estimates were 870 (no confidence intervals provided) in 1990 and 380 (CI 210–650) in 2008 [7]. Although the trend from both these estimates is downward, there are large discrepancies in the central figures for the maternal mortality ratio, albeit with overlapping confidence intervals. The estimates are also obtained from modelling processes, and whilst the latest empirical data from Nepal is included, such exercises have not always provided persuasive evidence for national stakeholders.

This paper is set in the context of an urgent need to have robust country estimates. By using demographic and health surveys, studies of maternal mortality and routine health service data, the analysis provides insights on the maternal mortality trend in Nepal. This study was commissioned:

- to test, using existing data, the plausibility and robustness of the reported decline in maternal mortality;
- to identify, using existing data, the driving factors in the decline of maternal mortality since 1997.

## Methods

### Plausibility of decline in maternal mortality

Two indicators of maternal mortality levels are used in this analysis. The maternal mortality ratio (MMratio) is the number of maternal deaths per 100,000 live births. It measures obstetric risk, which is the risk of dying of pregnancy related causes once pregnant. The second indicator is the maternal mortality rate (MMrate), which is expressed per 1,000 women aged 15–49 years. It provides an indication of the risk of maternal death in the adult female population and encompasses both the risk of being pregnant and the risk of dying once pregnant.

The national estimates used are the two NDHS from 1996 and 2006 [2], [4]. A reduction in the MMratio has previously been assumed based on the non-overlapping confidence intervals, but this was tested using a Poisson distribution and chi-squared test. Trends in sub-national mortality data were examined in NDHS. The timings of the mortality estimates were also examined.

The MMMS was carried out between 2008 and 2009 in eight districts and provides estimates of maternal mortality [6]. We used uncertainty modelling using WinBUGs software [9] to derive a nationally-representative 95% confidence interval for the MMratio based on the estimates from the eight districts.

The NDHS and the MMMS used different methods to measure maternal mortality. The NDHS asked women about the deaths of their sisters and identified maternal deaths from the timing of the death in relation to pregnancy [5]. The MMMS used a surveillance system where community informants reported births and deaths of women of reproductive age. This was followed by a verbal autopsy, which interviewed people regarding incidents and circumstances around the time of death to identify whether a death was pregnancy related [6]. The NDHS used nationally representative samples [2], [4]. The MMMS produced maternal mortality estimates for the eight districts which were selected to represent the different administrative regions and topological zones in Nepal [6].

### Assessing the impact of possible drivers on the observed decline in maternal mortality

Four hypotheses were examined as possible drivers of the decline in maternal mortality:

1. reduction in fertility rates;
2. reduction in high-risk pregnancies;
3. improvement in the coverage of care; and
4. societal trends.

The data were not available to allow a statistical examination of drivers of trends in maternal mortality using predictors and mortality in the same dataset, so alternative analyses were performed to gain some insight.

Estimates of MMratio and MMrate from the eight districts surveyed in the MMMS 2008 were combined with district level indicators from NDHS 2006. Potential drivers of the variation in maternal mortality between districts were identified by regressing each indicator against MMratio and MMrate. The amount of variation in mortality explained by each factor was measured by the value of the coefficient of determination, R^2^.

Further evidence was gathered by examining sub-national trends in mortality using both NDHS and MMMS data. Using NDHS, mortality estimates were produced for rural and urban areas, and the three development zones. Urban areas and the mountain zone were not associated with sufficient maternal deaths to allow meaningful estimates.

Analysis was also conducted employing the ‘familial assumption’ that sisters are likely to share certain characteristics [10]. The trend in maternal mortality (based on data from sisters of respondents) was adjusted for the education, poverty status, and rurality of the respondents. In this way, the contribution of changes in these characteristics to the observed trend in maternal mortality could be estimated.

Lastly, trends in the distribution of delivery care (professional attendance, facility delivery and Caesarean section) were examined. The two NDHS in 1996 and 2006 were used. Professional attendance was taken to mean delivery by a doctor, midwife or nurse.

## Results

### Plausibility of decline in maternal mortality

#### National maternal mortality estimates

**Figure 1:**
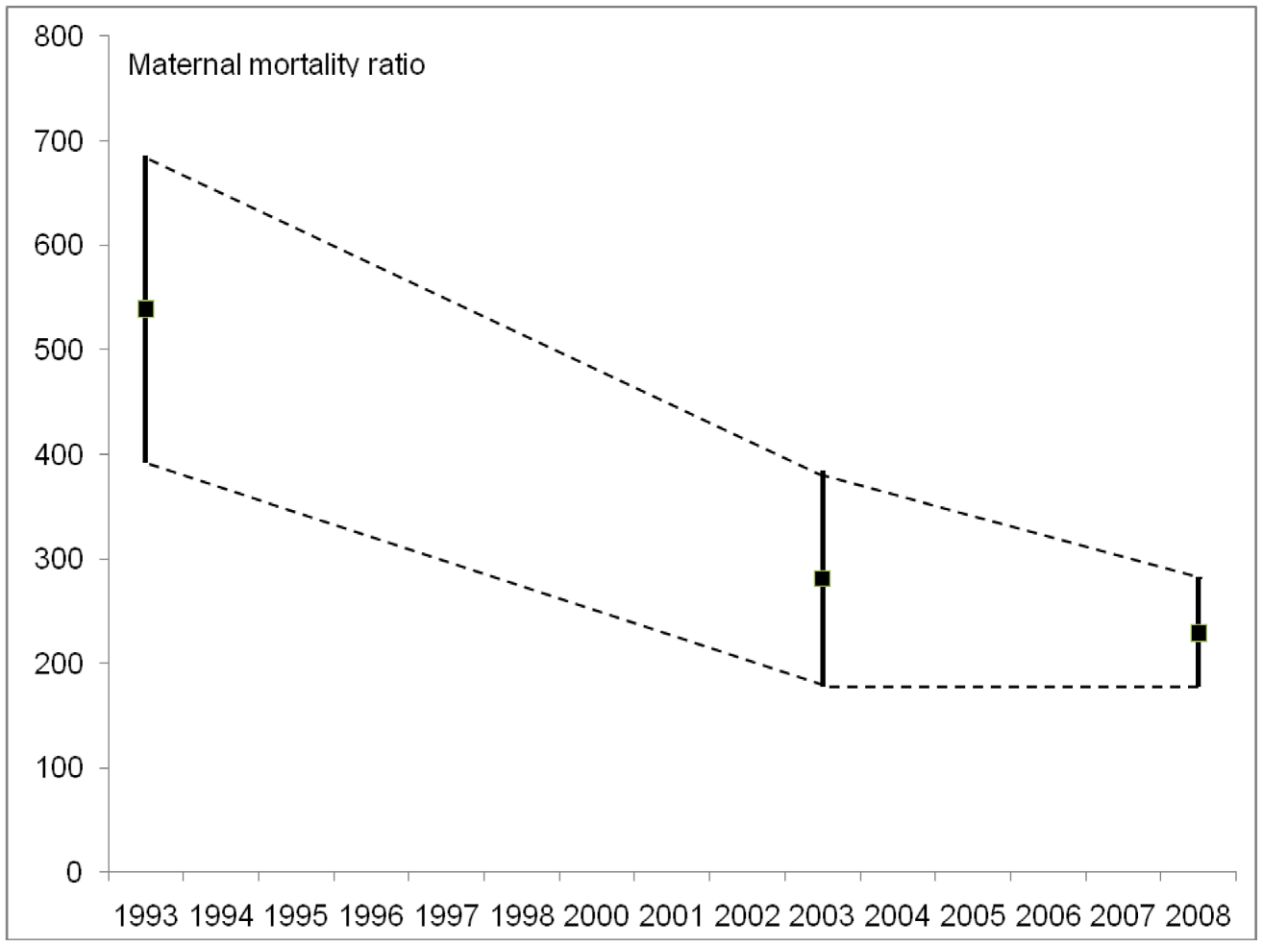
Estimates of maternal mortality with 95% confidence intervals, Nepal.

There is evidence to support a decline in maternal mortality since 1993, but there remains uncertainty about the size and temporal pattern of any change. The decline in MMratio measured by the two NDHS is just statistically significant. Data on mortality in both NDHS were collected for the preceding seven years, so the timing of the estimates is therefore not 1996 and 2006 (the years of the surveys) but approximately 3.5 years prior to these dates i.e. 1993 and 2003 (see Figure 1). Midpoints of the NDHS estimates show a decline of maternal mortality ratio from 539 to 281 per 100,000 live births between 1993 and 2003.

The nationally-representative 95% confidence interval calculated for the MMratio from the MMMS 2008 eight district data of 229 was 283 to 177 per 100,000 live births. The estimate is similar to that found by the 2006 NDHS but uses a different method to provide the maternal mortality estimate, so is supportive of a relatively low current level. The confidence intervals between the NDHS 2003 and MMMS 2008 points overlap, suggesting that any difference between the two levels may not be statistically significant.

Opinions differ on whether trends can be inferred from maternal mortality estimates generated using different methods – as in the case of the NDHS and MMMS. Figure 1 shows the three estimates along with the boundaries for possible pathways of the observed decline that are consistent with the uncertainty in the estimates. Joining the mid-points of the confidence intervals indicates a steady decline in MMratio between 1993 and 2008, but some of the alternative patterns include: no decline between 1993 and 2003 followed by a sharp decline between 2003 and 2008; or a sharp decline between 1993 and 2003 followed by a slight rise between 2003 and 2008. The overall trend supports a decline in maternal mortality, even if the actual levels lack precision.

#### Sub-national mortality data

Table 1 shows estimates of MMratio from NDHS at a national level and also for rural areas, hill and terai (the flat plains of Nepal) development regions. Urban and mountain areas are not shown due to insufficient numbers of deaths. The sub-national changes in MMratio are of similar magnitude and direction to those observed nationally, and in the terai the differences are statistically significant. This consistency between areas strengthens the evidence for a decline. The terai levels were higher than the other areas initially, but the 2006 levels are similar in each area. The estimates are determined by the respondents who are sisters of the women who died.

**Table 1:** National and sub-national maternal mortality ratios, Nepal.

	Maternal mortality ratio		
Area	1993^a^	2003^a^	Difference(95% Confidence interval)
All*	539	281	258 (91,507)
Rural	513	284	228 (−8,465)
Hill	408	287	121 (−180,423)
Terai (plains)*	645	284	361 (36,686)

Data source: Nepal demographic and health surveys 1996 and 2006.

a Timing of the estimates is 3.5 years prior to the dates of the surveys.

*Denotes statistical significance of difference at less than 5%.

### Drivers of maternal mortality decline

Using MMMS 2008 data, the district-level analysis reported in Table 2 suggests that the General Fertility Rate (GFR), Human Development Index (HDI), Gender Empowerment Measure (GEM), the percentage of anaemic mothers, met need for emergency obstetric care and age at first birth each explained more than 10% of the district variation in MMratio. The HDI is an index used by the United Nations to assess the well-being of a population and includes factors such as poverty, education and life expectancy [11]. The GEM is used to measure aspects of gender equity such as the level of women's participation in political and economic decision making [12].

**Table 2:** Associations between maternal mortality rate or ratio and potential drivers of variation, Nepal.

Drivers of variation^a^	Maternal mortality ratioβ and R^2^	Maternal mortality rateβ and R^2^
% Professional delivery care	38** 0.007	−0.173** 0.098
% Hospital delivery	8.4** 0.0003	−0.174** 0.085
% Health facility delivery	93** 0.05	−0.086** 0.026
% Emergency obstetric care facility delivery	0.87** 0.08	0.0001 0.000
% Caesarean section delivery	0.760** 0.006	−0.003** 0.057
% Met need for emergency obstetric care	0.942** 0.22	0.0006** 0.06
Mean birth order	−28** 0.02	0.026** 0.013
Mean wealth quintile	5.8** 0.008	−0.015** 0.035
Mean total children born	−12** 0.004	0.035** 0.025
Mean age at first birth	−44** 0.178	0.023** 0.033
Mean haemoglobin	−2.50** 0.198	−0.0008** 0.014
% anaemia moderate/severe	118** 0.38	−0.095** 0.163
% any birth preparation	−4.38** 0.0004	0.0048** 0.0003
Human Development Index (regional)	−713** 0.283	−1.176** 0.51
Gross Domestic Product (regional)	5.48 0.000	0.071** 0.002
Gender Empowerment Measure (regional)	−575** 0.35	−0.714** 0.356
General Fertility Rate	1.12** 0.109	0.003** 0.613

Data source: Maternal mortality and morbidity survey, 2008 weighted by number of live births. β is the slope of the regression line and R^2^ the coefficient of determination.

**Denotes statistical significance of difference at less than 1%.

a Drivers are of district variation unless stated otherwise, depending on availability of data.

The amount of variation in mortality explained by a factor was measured by the value of R^2^, so for example GFR explains 61.3% of the variation in MMrate. Their combined effect could not be estimated due to the small number of districts surveyed. This analysis provided the basis upon which a number of hypotheses are proposed.

#### Hypothesis 1: Reduction in fertility rate

**Figure 2:**
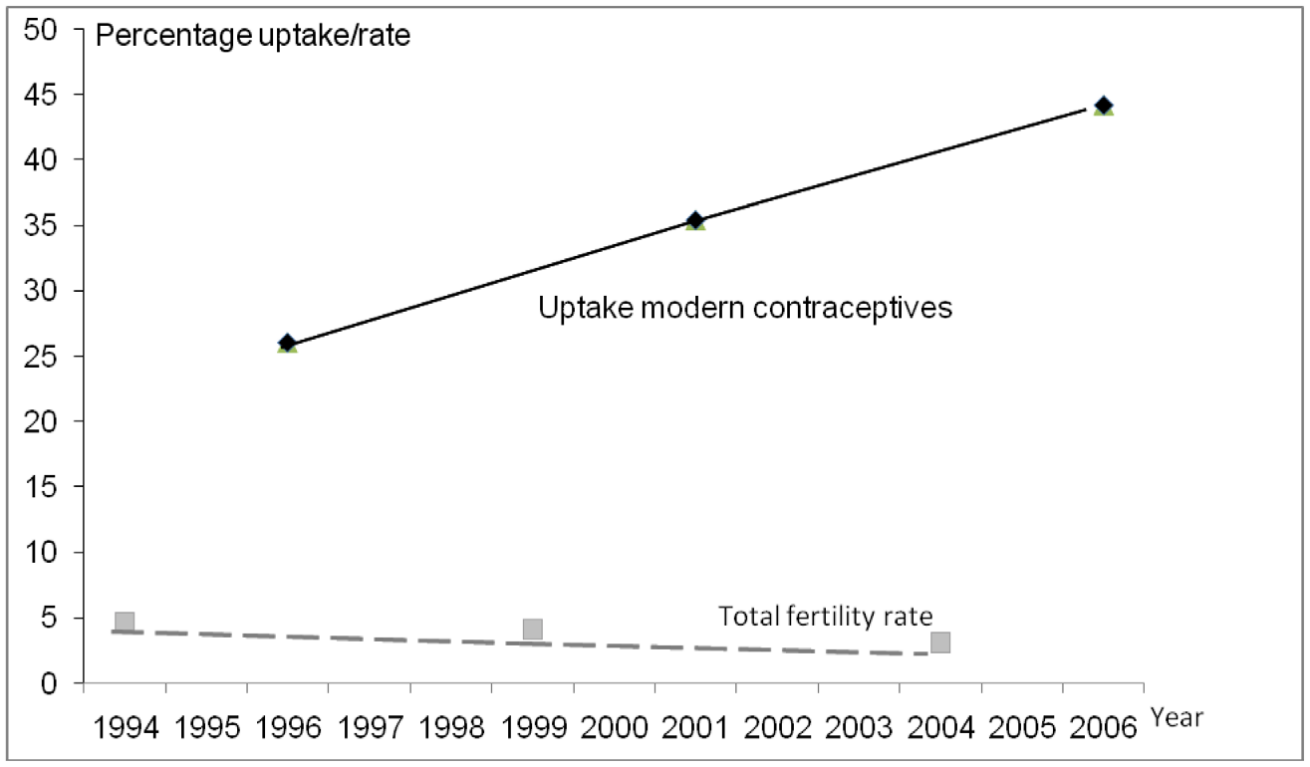
National fertility rate and contraceptive coverage, Nepal.

**Figure 3:**
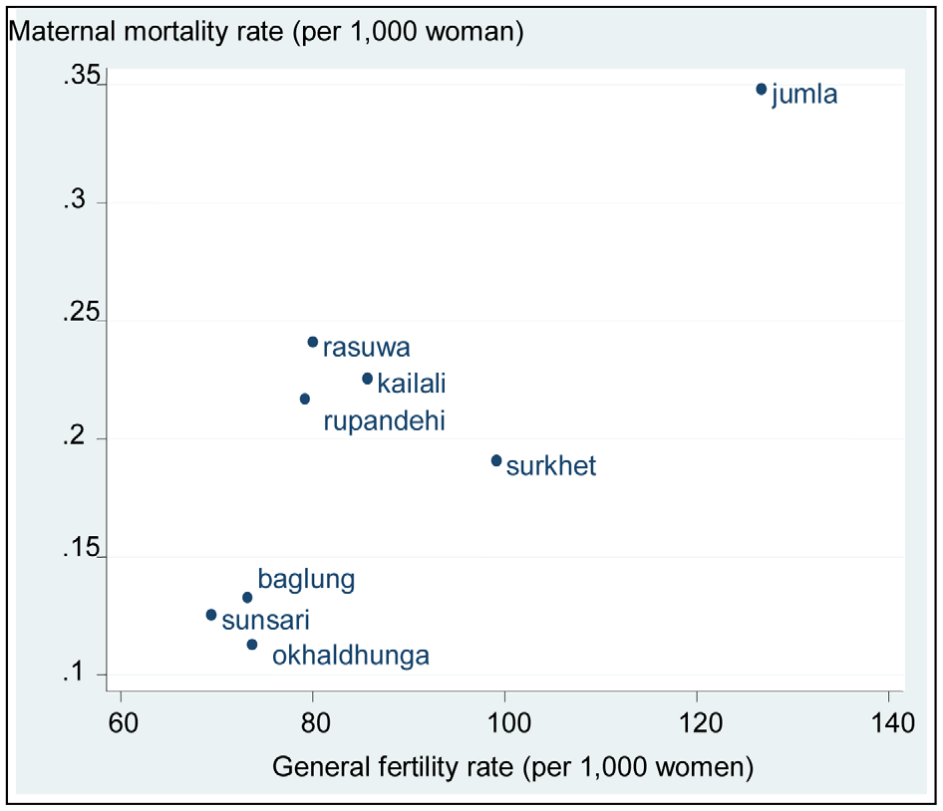
Maternal mortality rate by fertility for eight districts in Nepal.

The NDHS reported that fertility declined from 4.6 births per woman in 1994 to 3.1 in 2004. This was based on fertility three years prior to the survey (for example, the NDHS 1996 reference period was 1993–1995 so the estimate was placed at the mid-point of 1994). Figure 2 illustrates the drop of one and a half births per woman in ten years. The decline in fertility was greater in the five years between 2001 and 2006 than between 1996 and 2001. The percentage of women using modern contraceptive methods showed a corresponding steady increase. The regression analysis reported in Table 2 relevant to this question is shown in Figure 3; it illustrates the high level of correlation between GFR and MMrate.

Table 3 shows estimates of MMrate at a national level and also for rural, hill and terai areas. The national MMrate has fallen by around two-thirds, compared with MMratio estimates shown earlier (Table 1) which only halved. Such patterns are expected in a situation where fertility is declining. The greater effect is seen in the MMrate as fewer women are exposed to the risks of pregnancy, but there is also a reduction in MMratio as the risk profiles of mothers change – fewer deliver at the extremes of age; birth intervals become longer; and occurrence of high parity births fall.

**Table 3:** National and sub-national maternal mortality rates, Nepal.

	Maternal mortality rate		
Area	1993^a^	2003^a^	Difference(95% Confidence interval)
All*	0.872	0.333	0.54 (0.2,0.9)
Rural*	0.882	0.361	0.52 (0.2,0.9)
Hill	0.666	0.315	0.35 (−0.1,0.8)
Terai* (plains)	1.007	0.339	0.67 (0.2,1.1)

Data source: Nepal Demographic and Health Surveys 1996 and 2006.

a Timing of the estimates is 3.5 years prior to the dates of the surveys.

*Denotes statistical significance of difference at less than 5%.

#### Hypothesis 2: Reduction in high-risk pregnancies

**Figure 4:**
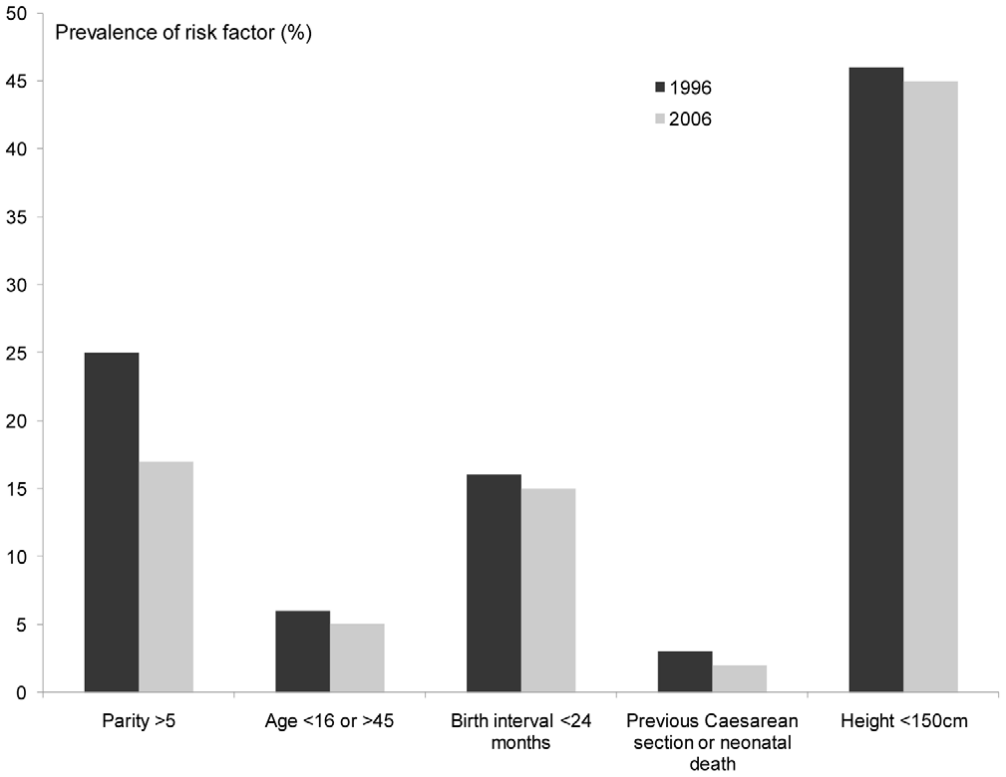
Changes in pregnancy related risk factors over time, Nepal.

The percentage of anaemic mothers and mean age at first birth also explained more than 10% of the district variation in maternal mortality ratio in the MMMS. Other risk factors linked to maternal mortality can be measured using NDHS, but there has been very little change in the prevalence of extremes of age for childbirth (<16 or >45 years), short birth interval (<24 months), history of Caesarean section or neonatal death and short stature (<150 cm). The only risk factor that has shown any significant decline is grand-multiparity (>5 deliveries) which is in line with the fertility drop (Figure 4). Fertility has declined in every age group over the past 10 years, with larger declines seen in the older age cohorts.

#### Hypothesis 3: Improvement in coverage of care

**Figure 5:**
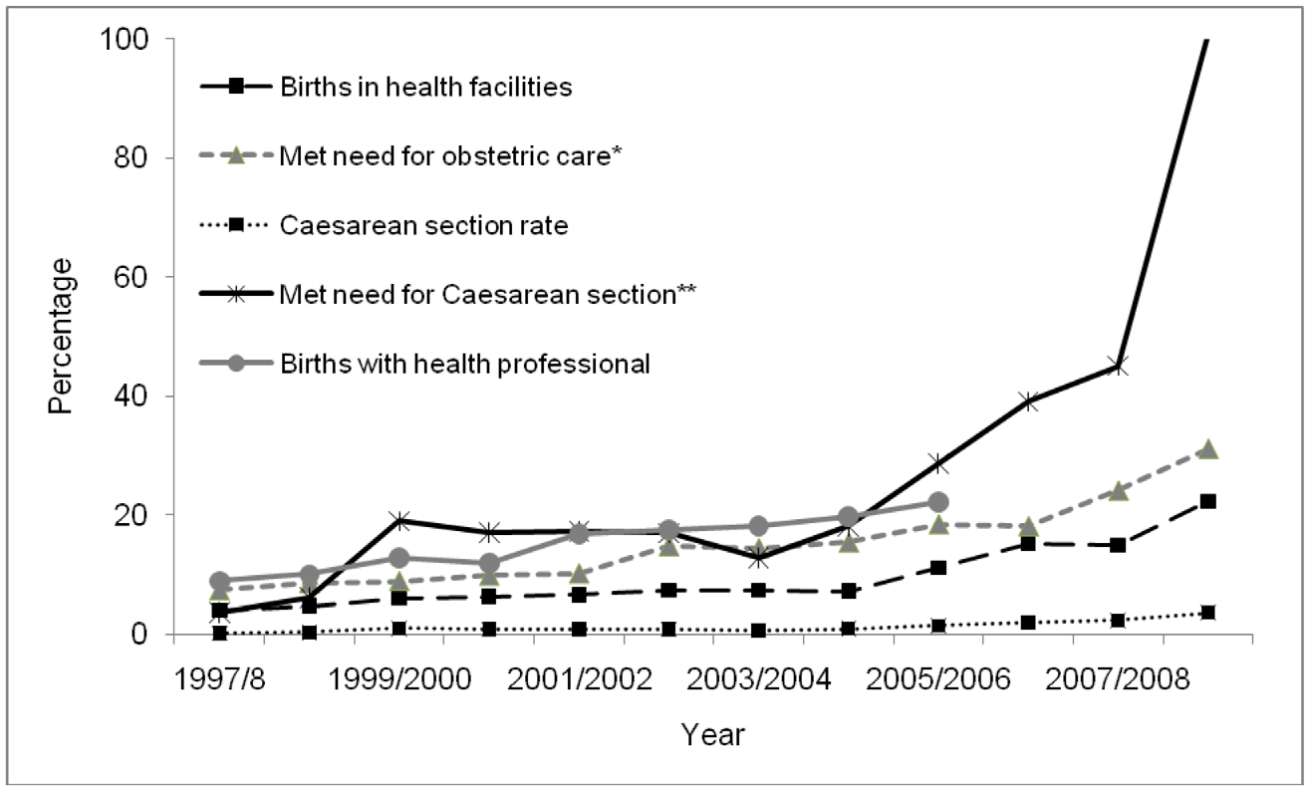
Trends in obstetric care indicators for selected districts, Nepal.

**Figure 6:**
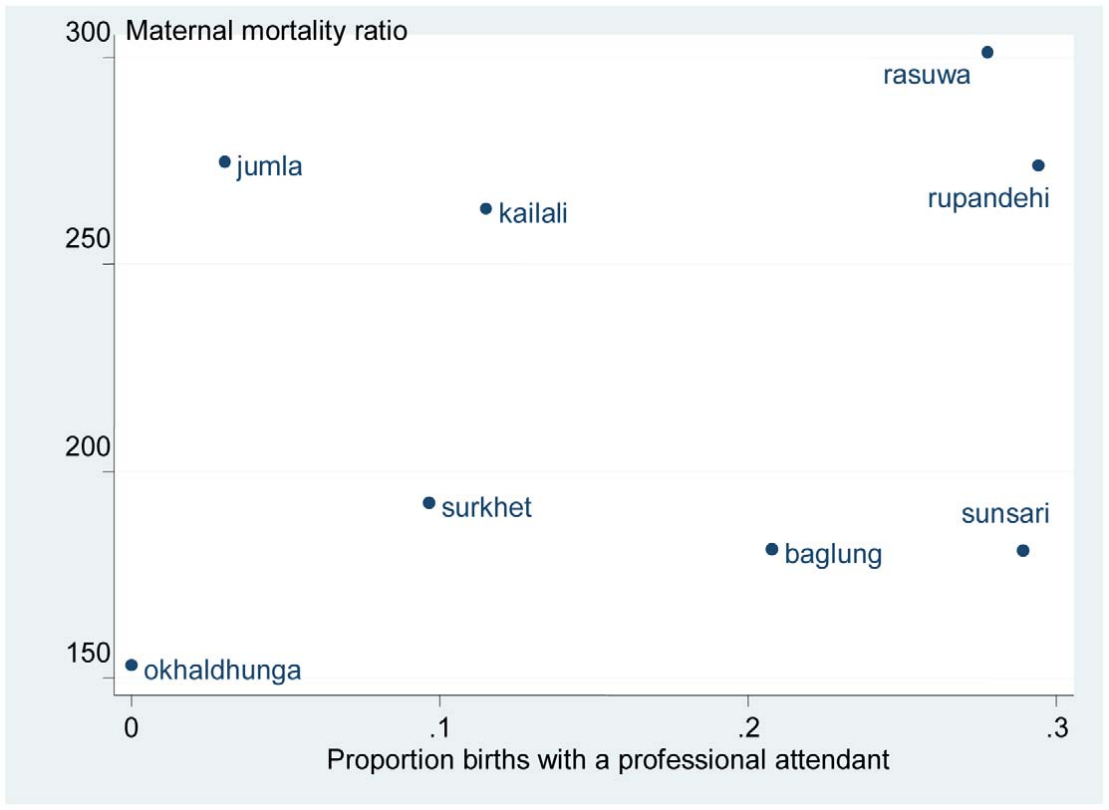
Estimates of maternal mortality ratio by births with a health professional for eight districts in Nepal.

Data from Table 2 also suggest that met need for emergency obstetric care (the proportion of complications seen, out of an expected 15% of pregnancy complications), explains district variation in maternal mortality. Figure 5 illustrates the trends seen in some key indicators of obstetric care. Met need for emergency obstetric care and Caesarean section have both increased over the period of interest, although most of the change occurred after 2005. As the timing of the observed change in MMratio is unclear, it is not possible to know whether the changes in indicators of coverage of care could have driven the decline. The most likely pattern of change in MMratio (from joining mid-point estimates) is a steady decline between 1993 and 2008. Only the proportion of births with a professional has a similar pattern; the other indicators remain fairly static until 2004/5 and then show an increase. However the evidence is not consistent because the relationship between MMratio and skilled birth care was weak in the district analysis (Table 2 and Figure 6), explaining less than 1% of the variation in maternal mortality between districts.

**Figure 7:**
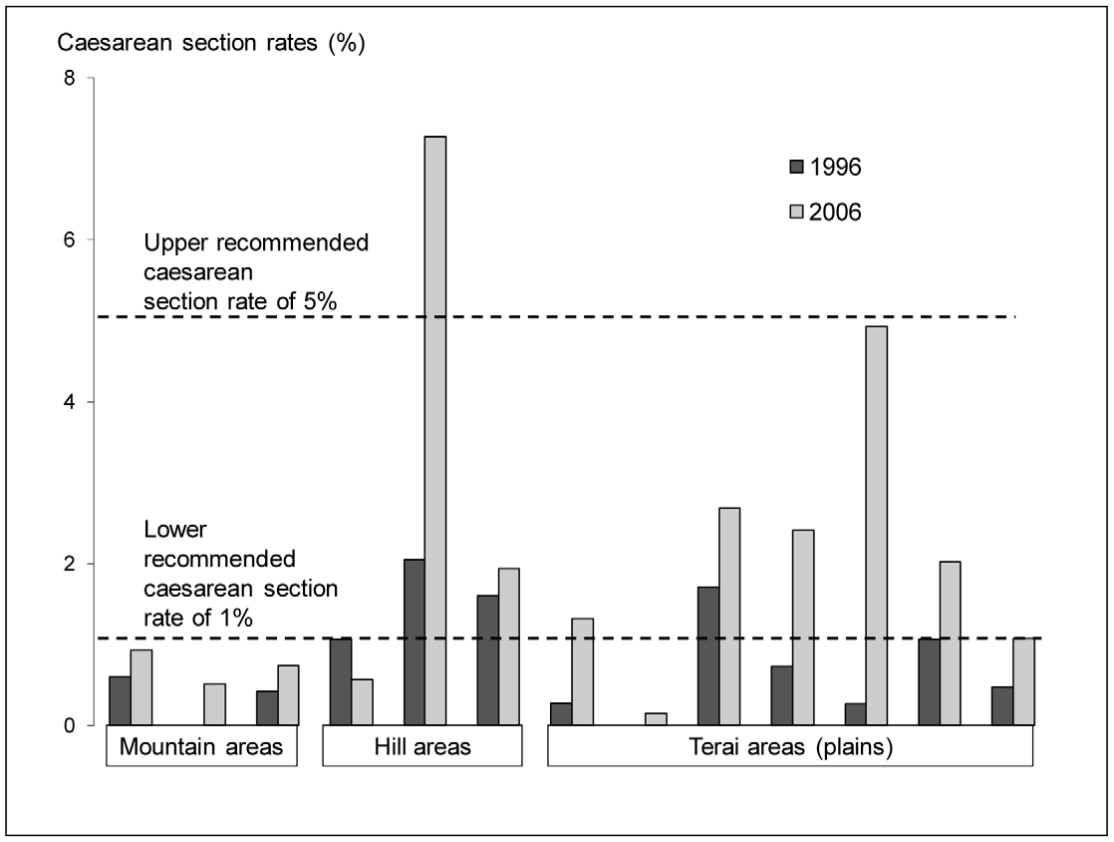
Regional estimates of changes in Caesarean section rates, Nepal.

Caesarean section can be an important life-saving intervention and the national level has increased from 1% to 3%. The distribution of that increase and to what extent it meets obstetric needs will determine how effective it is in reducing maternal mortality. Figure 7 shows that for the first time two regions have reached levels of 5% and in several others, levels of 1%. Caesareans section rate of between 1% and 5% have been suggested as necessary to save the lives of mothers and their babies [13].

#### Hypothesis 4: Societal trends

The HDI and the GEM also each explained more than 10% of the district variation in maternal mortality ratio (Table 2). The HDI combines dimensions of life expectancy, education and standard of living, The GEM measures inequalities between men and women in political and economic participation.

Table 4 shows that changes in the education and wealth of women giving birth between the two surveys were found to explain part of the trend in MMrate; changes in where women live in terms of rurality and ecological zone did not seem to have affected the trend. The MMrate in 2003 (from the 2006 NDHS) was used as the reference, so the incidence rate ratio represents the trend in MMrate, and the unadjusted estimate shows that the level in 1993 was 2.69 times greater than the estimate for 2003. Adjusting for education and wealth reduces the size of the incidence rate ratio, meaning that changes in these factors explain part of the trend observed in MMrate. This analysis was not taken further because it relies on an assumption that sisters share characteristics, and this is not always true, for example, sisters may not necessarily share delivery care characteristics.

**Table 4:** Trend in Nepal's national estimate of maternal mortality rate, adjusted for respondent characteristics using Poisson regression.

Adjusted for:	Incidence rate ratio (95% Confidence interval)
(reference)^a^	2.69 (1.8,4.0)**
education	2.15 (1.4,3.3)**
wealth quintile	1.92 (1.2,3.0)*
rurality	2.61 (1.8,3.8)**
eco zone	2.72 (1.9,4.0)**

Data source: Nepal demographic and health surveys 1996 and 2006.

a maternal mortality rate from the later survey was used as the reference, so the incidence rate ratio in the earlier survey was 2.69 times greater.

**denotes statistical significance at p<0.001;

*p<0.01.

Although Table 3 showed that the MMrate has fallen by about two thirds, an analysis of non-maternal mortality in women of reproductive age shows a reduction of only 1.6 (Table 5), obtained by dividing the difference between the non-maternal and maternal mortality rates of 1993 and 2003, Tables 5 and 3, (3.84-0.87)/(2.12-0.33). There is thus a steeper decline in maternal compared with non-maternal mortality, which suggests that the fall observed in maternal mortality is not explained simply by general improvements in health, providing evidence for the contribution of specific interventions directed at decreasing maternal mortality.

**Table 5:** Non-maternal mortality, women of reproductive age, Nepal.

	Non maternal mortality, women of reproductive age		
Area	1993^a^	2003^a^	Difference (95% confidence intervals)
All*	3.84	2.12	1.72 (0.9,2.5)
Rural*	3.97	2.27	1.71 (0.9,2.6)
Hill*	3.50	2.00	1.50 (0.4,2.6)
Terai* (plains)	4.45	2.24	2.20 (1.1,3.7)

Data source: Nepal demographic and health surveys 1996 and 2006.

a Timing of the estimates is 3.5 years prior to the dates of the surveys.

*Denotes statistical significance of difference at less than 5%.

## Discussion

The plausibility of the maternal mortality decline in Nepal is supported by several sources. A statistically significant decline in maternal mortality was demonstrated since 1993 using the two NDHS. The 2008 MMMS estimate of maternal mortality supports a relatively low current level and implies that mortality has fallen at some time in the past. Sub-national analysis mirrors the national decline in NDHS with valid results from rural, hill and terai regions.

However, with two comparable points, the evidence for a trend cannot be conclusive. There are considerable limitations in the data we have available and of these, the most important relate to the underestimation of numbers of deaths, which imply that MMratio is likely to be higher than reported. The NDHS is known to under-report maternal mortality in household surveys [14]. The estimates available are based on relatively small numbers of deaths, for example, there were only 39 deaths found in the NDHS 2006. The mortality data is collected from respondents about their sisters, so sub-national estimates of MMratio need to be interpreted carefully as sisters may live in different regions.

A number of possible factors may explain the reduction in maternal mortality. The observed fertility decline is likely to be an important driver of change and occurred over the same period of time as maternal mortality reduction. We found a reduction in high risk pregnancies, possibly in relation to the fertility decline. Societal changes may also explain part of the decline. Education and wealth quintile explained part of the decline in maternal mortality and this was established in our analysis using the familial assumption [10], that sisters will have the same socio-economic characteristics. In addition, GFR, HDI, GEM, anaemia and age at first birth were the most important factors we found to explain variation in maternal mortality at district level. It may be that maternal mortality is falling because women are enjoying better health. Healthier women may be physically better prepared to withstand the dangers of pregnancy, thereby contributing to maternal mortality reduction. However, this may not be the only explanation for observed reductions. Other factors will contribute such as coverage of care or its quality.

The relatively steep decline in maternal mortality compared with non-maternal mortality however suggests that the fall observed is not explained simply by general improvements in health. Specific changes in obstetric care are observed and delivery care (health professional, health facility, met need for emergency obstetric care and Caesarean section) are all increasing. The data are not available to determine whether improvements in care occurred at the same time as the observed fall in mortality.

Our analysis of the drivers of change is largely ecological. It is difficult to infer causality from such analysis and with the small numbers of districts included in the MMMS. The correlations between potential drivers of variation and district maternal mortality indicators are too weak to permit national maternal mortality to be modelled.

It is not possible to examine drivers of trends in maternal mortality statistically, as predictor and mortality data were not available in the same dataset. Potentially important factors like abortion care, birth preparedness, prevalence of anaemia, or women's empowerment could not be examined further due to lack of data. However, in Nepal, improvements in abortion care did not happen until 2005, so are probably not linked to the maternal mortality decline.

A comparison between the situation in Bangladesh and Nepal is of interest. Both show declines in maternal mortality despite low rates of deliveries with health professionals. The halving of maternal mortality in government service areas in Bangladesh occurred over 30 years, but Nepal has experienced the same change over 10 years. Fertility reductions, better education and improving wealth also occurred in Bangladesh during the time of the maternal mortality decline, although better abortion and emergency obstetric care seemed to have contributed [15]. Other social changes relate to decision making power, ability to independently generate income and participation in the labour force could have contributed and warrant further exploration. The weak explanatory power of deliveries with health professionals in reducing maternal mortality in both Nepal and Bangladesh is notable, but data availability in Nepal did not allow us to investigate related factors such as quality of care provided or timing of maternal death. This may be another area for future research using, for example, assessment of hospital deaths. Antenatal care uptake has increased in both countries, but the pathway through which it might reduce maternal mortality is less obvious, so it was not included in our analyses.

When the NDHS and MMMS findings were released in Nepal, the decline in maternal mortality was celebrated as evidence of success related to the Nepal government's progressive policies on maternal and newborn health, equity and poverty; and their implementation into practice with assistance by development partners. Our objective was to provide a rigorous and objective appraisal of the plausibility and drivers of change. Given the limitations faced in obtaining adequate data for a conclusive assessment, what was the value of such an exercise?

Nepal is planning a third DHS maternal mortality study in 2016. This study will mark a crucial point in the history of Nepal's efforts to reduce maternal mortality. If a continued reduction in maternal mortality is observed, that will be good news all round. However, if the maternal mortality estimate does not support a continued downward trend, the findings presented in this paper at least provides insights that the magnitude of change may not be as large as what might be otherwise anticipated. Such forewarning may prevent disillusionment and diversion of interests to other programmes which can show successes more easily and with greater certainty. It will also allow time to prepare for the communication of NDHS 2016 findings, irrespective of whether maternal mortality reduction is clearly demonstrated or not.

The lack of good data to inform trends in maternal mortality reduction and its determinants underscores the need to plan for improved means to track maternal mortality in Nepal. If more robust identification of causes for the decline in maternal mortality is considered a priority, there may be a need for considering the establishment or expansion of an existing surveillance site in Nepal, which would allow observations of factors contributing to the changes, as has been conducted in Matlab, Bangladesh [15] although this may be a costly endeavour. A longer term strategy would be to invest in building up a reliable register of births and deaths. The potential association found between anaemia and district variation in maternal deaths is intriguing and especially relevant to other South Asian countries. The data available in this study did not allow further conclusions to be drawn. Large, good quality studies which investigate clinical outcomes and adverse effects are called for [16], [17].

As little is known about why the decline in maternal mortality has occurred (and in anticipation of a demonstrated continued decline) a historical case study exploring factors which may have caused the decline is of interest and could provide a contemporary and new ‘success story’ similar to the widely referenced successes of countries like Malaysia, Thailand and Sri Lanka. Such an exploration should investigate and bring together what has already been documented on historical changes in gender empowerment, community mobilisation, migration, transportation, communication systems and well as general health and health services. This would bring a greater understanding of why and how maternal mortality reduction can be achieved in settings where terrain, poverty and remoteness remain very real challenges.
